# 726 Enteral Tube Clogging and Medication Administration Techniques

**DOI:** 10.1093/jbcr/irad045.201

**Published:** 2023-05-15

**Authors:** Esther Rathjen, Grace Oehlertz

**Affiliations:** CHI Health St Elizabeth, Lincoln, Nebraska; CHI Health St. Elizabeth, Lincoln, Nebraska; CHI Health St Elizabeth, Lincoln, Nebraska; CHI Health St. Elizabeth, Lincoln, Nebraska

## Abstract

**Introduction:**

Many burn patients require long term enteral tube placement. Preventing clogging is essential for patient comfort as well as for preventing pauses in nutritional supplementation required for wound healing. While many studies have been done to measure nursing compliance with policies intended to prevent tube clogging, no research has been done on whether administering pills via enteral tube one at a time with a flush between actually prevents clogs. The purpose of this pilot study was to see if small bore enteral tubes clog more often when medications are given all at once in solution compared to when given individually with a flush between pills.

**Methods:**

Two enteral tubes were connected to continuous tube feed by pump. The ends were placed in a bucket. Medications were crushed and administered through the tubes. Pills were given through the control one at a time with a flush between, and through the experimental tube all at once in solution. Water flushes were performed before and after medication administration and care was taken that all other conditions were the same between groups. Each med pass was done approximately 24-48 hours apart. If more than gentle, continuous pressure was required to administer the medications and flushes the tube was considered clogged.

**Results:**

Medications were given through both tubes 36 times. Of the 72 administrations there were 10 clogs, 5 to the control tube and 5 to the experimental tube.

**Conclusions:**

Results of this pilot study suggest that individually crushing and administering each pill when giving them through an enteral tube does not reduce the incidence of clogs. Considering the time demand and potential for increased fluid intake using this practice we propose giving all medications together in solution as a safe alternative. Especially in the burn population where accurate intake is vital for calculating fluid resuscitation, having a consistent protocol is essential. While the sample size of this study is too small to definitively say there is no difference between medication administration techniques, issues related to fluid and electrolyte balance as well as nursing compliance with policy should be considered when deciding to maintain the current standard of practice which has no basis in evidence

**Applicability of Research to Practice:**

Administering medications through enteral tubes is a frequent nursing intervention. If applied to practice, these findings would change how nurses are taught to administer medications as well as change the process by which care is delivered on a daily basis.

